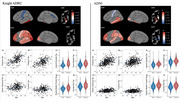# Age‐ and sex‐effects on tau PET binding in the absence of beta‐amyloid pathology

**DOI:** 10.1002/alz70856_098038

**Published:** 2025-12-24

**Authors:** Diana A Hobbs, Brian A. Gordon, Shaney Flores, Sarah J. Keefe, Nicole S. McKay, Julie K. Wisch, Russ C. Hornbeck, Erin E. Franklin, Chengjie Xiong, John C. Morris, Richard J. Perrin, Beau Ances, Yi Su, Hillary D. Protas, Tammie L.S. Benzinger

**Affiliations:** ^1^ Washington University School of Medicine, St. Louis, MO, USA; ^2^ Washington University School of Medicine, Saint Louis, MO, USA; ^3^ Washington University in St. Louis, St. Louis, MO, USA; ^4^ Washington University in St. Louis, School of Medicine, St. Louis, MO, USA; ^5^ Washington University in St. Louis School of Medicine, St. Louis, MO, USA; ^6^ Washington University School of Medicine in St. Louis, St. Louis, MO, USA; ^7^ Department of Neurology, Washington University in St. Louis School of Medicine, St. Louis, MO, USA; ^8^ Banner Alzheimer's Institute, Phoenix, AZ, USA

## Abstract

**Background:**

We characterized primary age‐related tauopathy (PART) in individuals without elevated amyloid levels and explored whether levels of this protein differed by sex.

**Method:**

Tau burden was quantified using PET imaging (18F‐flortaucipir) in cognitively unimpaired participants with amyloid burden below pathological levels (PET SUVR≤17.15, determined via GMM) from the Knight ADRC (*n* = 251, age: 45‐91) and ADNI (*n* = 458, age: 50‐94) cohorts. Statistical models were deployed to assess the influence of age, sex, race, and subthreshold amyloid burden on regional tau distribution.

**Result:**

Significant positive associations between tau PET binding and age occurred in 7 (Knight ADRC_1_) and 13 (ADNI_2_) regions, with the largest effects seen in the putamen (t_1_=9.40; t_2_=10.50) and pallidum (t_1_=8.36; t_2_=7.39). Elevated tracer uptake in females was present in 19 (Knight ADRC_1_) and 12 (ADNI_2_) regions, but most prominently in the lateral occipital (t_1_=6.67; t_2_=4.31), pars orbitalis (t_1_=4.93; t_2_=4.75), rostral middle frontal (t_1_=4.73; t_2_=4.85), and the frontal pole (t_1_=4.59; t_2_=5.31). No statistically significant influence of race or subthreshold amyloid burden was observed for either cohort.

**Conclusion:**

Tau PET is sensitive to age‐related tau accumulation and reveals a distribution pattern consistent with the neuropathological features of PART. Women consistently show higher tracer uptake across multiple brain regions suggesting previously reported increases in tau‐burden for females is not merely an implication of AD pathology, but may reflect a widespread, intrinsic phenomenon. Notable age‐associated increases in the basal ganglia are areas known for off target binding. A deeper understanding of the influences of age and sex on tracer uptake is essential for accurate interpretation of imaging data, particularly in distinguishing normal aging from disease related changes.